# TULA-Family Regulators of Platelet Activation

**DOI:** 10.3390/ijms232314910

**Published:** 2022-11-28

**Authors:** Satya P. Kunapuli, Alexander Y. Tsygankov

**Affiliations:** Sol Sherry Thrombosis Research Center, Lewis Katz School of Medicine, Temple University, Philadelphia, PA 19140, USA

**Keywords:** platelet, signaling, phosphorylation, Syk, ITAM, hemITAM, TULA-2

## Abstract

The two members of the UBASH3/TULA/STS-protein family have been shown to critically regulate cellular processes in multiple biological systems. The regulatory function of TULA-2 (also known as UBASH3B or STS-1) in platelets is one of the best examples of the involvement of UBASH3/TULA/STS proteins in cellular regulation. TULA-2 negatively regulates platelet signaling mediated by ITAM- and hemITAM-containing membrane receptors that are dependent on the protein tyrosine kinase Syk, which currently represents the best-known dephosphorylation target of TULA-2. The biological responses of platelets to collagen and other physiological agonists are significantly downregulated as a result. The protein structure, enzymatic activity and regulatory functions of UBASH3/TULA/STS proteins in the context of platelet responses and their regulation are discussed in this review.

## 1. Novel Family of Atypical Protein Tyrosine Phosphatases

The family discussed in this review is currently termed UBASH3 for the ubiquitin-associated (UBA) and Src-homology 3 (SH3) domain-containing gene. The protein product of UBASH3A is also called STS-1 for the suppressor of TCR signaling, CLIP4 for the Cbl-interacting protein and TULA (or TULA-1) for the T-cell ubiquitin ligand, while the protein product of UBASH3B, originally designated p70, is also called STS-1 and TULA-2 [1,2,3,4,5,6,7]. The terms TULA-1 and TULA-2 will be used in this review solely for the sake of consistency, even if a respective original paper uses a different name. Extensive discussions of all TULA-related findings, including platelet-unrelated ones, which are not a direct focus of this review, can be found in previous reviews on this topic [6,7,8,9,10].

**Figure 1:**
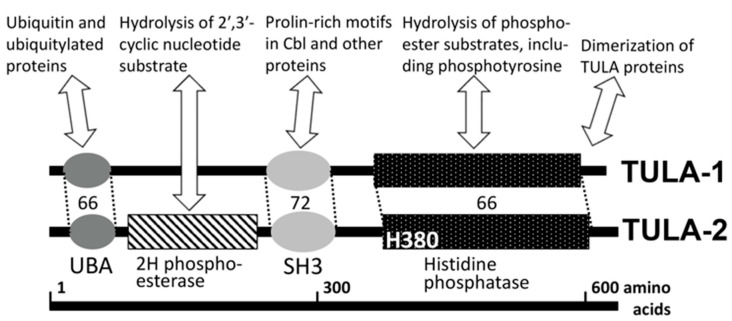
TULA-family protein structure, domains and major interactions. Major functional domains of UBASH3/STS/TULA proteins are shown, including ubiquitin-associated domain (UBA), Src-homology domain 3 (SH3) and histidine phosphatase (HP) domain. The 2H phosphoesterase domain has been identified in TULA-2. The degree of homology within major domains is shown as the percentage of similar (‘positive’) amino acid residues. Major interactions, including enzymatic activities, are outlined for various domains; most of them are characteristic of both family members. The key catalytic histidine of the histidine phosphatase domain is indicated (H380 in human TULA-2). The C-terminal sequence mediates dimerization of TULA proteins. See details in the text.

The structure of TULA proteins is conserved within the family and sports an unusual combination of functional domains [1,2,3,4] (Figure 1). One of them is a phosphatase domain containing a catalytic histidine residue (histidine phosphatase, or HP domain [11]), which confers phosphatase activity to TULA-family members [12]. The nature of the TULA-family HP domain sharply differentiates it from the typical protein tyrosine phosphatases (PTPs), whose key catalytic residue is cysteine [13,14]. The N-terminal half of TULA-family proteins contains UBA and SH3 domains, which interact with ubiquitin and proline-rich-motif-containing proteins, respectively [4,5,15,16], and appear to regulate various cellular processes [4,5,12,15,16,17,18,19,20,21] (see Figure 1). Finally, a domain exhibiting 2′-phosphodiestease activity has been recognized in the region between UBA and SH3 domains, although its physiological substrates remain unclear [22] (see Figure 1).

## 2. PTP Activities of TULA-Family Proteins and Their Possible PTP-Independent Functions

The structures of TULA-1 and TULA-2 are very similar, especially within the functional domains; this notion is apparent from sequence comparisons and structural studies [12,23,24,25,26]. However, the two family members are very different in their enzymatic activity; TULA-2 activity is much higher than that of TULA-1 [12,24,27]. Quantitative comparisons of activities have been mostly performed using small molecules and total cellular phosphotyrosine (pY)-containing protein [12,24,28] and also with recombinant full-length Syk [27]. The observed difference varies depending on the substrates and reaction conditions used but always remains profound. Thus, the difference in activity between TULA-2 and TULA-1 toward *p*-nitrophenyl phosphate (pNPP) is ~6000-fold, but for 3-O-methyl-fluorescein phosphate (OMFP), it is ~200-fold [24,28]. Furthermore, the difference between TULA-2 and TULA-1 activities toward pNPP is reduced at pH 5.0, where the maximal activity of TULA-1 is detected but still remains ~200-fold [24]. Likewise, the activity of TULA-1 toward total immunoprecipitated pY-proteins from T cells becomes detectable at pH 5.0, but the difference between TULA-2 and TULA-1 remains no less than two orders of magnitude [24].

These results are consistent with the finding that multiple pY-peptide substrates of TULA-2 have been identified in the course of random peptide library screening, while no substrates have been identified for TULA-1 in these experiments [29]. The substrates of TULA-2 found by screening were validated and further characterized using enzyme kinetic analysis with multiple synthetic pY-peptides [29], and this substrate specificity was confirmed for individual pY-sites of Syk, a bona fide substrate of TULA-2, both in reaction mixes and in platelets [27,29]. Notably, the HP domain of TULA-2 is sufficient for governing substrate specificity [29].

One may hypothesize that UBA-mediated interactions are important for the binding of TULA PTPs to their substrates, since many pY-containing proteins are ubiquitylated [30,31,32,33,34,35]. This hypothesis is supported by the accumulation of ubiquitylated pY-proteins in T-cell receptor (TCR)/CD3-stimulated mouse T cells lacking both TULA-1 and TULA-2 (double knockout, dKO) as compared to wild-type (WT) T cells [36] and by a decrease in the ability of TULA-2 to reconstitute WT signaling levels in dKO T cells as a result of the mutational inactivation of UBA [12]. However, UBA–ubiquitin binding is unlikely essential for TULA–substrate interactions, since the dephosphorylation of multiple non-ubiquitylated pY-peptides and pY-proteins by TULA PTPs has been shown [12,27,29] and since the HP domain of TULA-2 alone is sufficient to govern substrate specificity [29].

It should be noted that most studies with TULA-family proteins were conducted with mouse proteins. However, human TULA-1 and TULA-2 have been described as structurally similar and behaving comparably to their mouse counterparts [26]. As shown for mouse TULA-family proteins, human TULA-2 is substantially more active than human TULA-1, although some subtle differences are apparent between human and mouse TULA families [26]. Another significant finding of this study is that the PTP kinetics of full-length human TULA-2 and its HP domain are reasonably similar, confirming the conclusion that TULA HP domains can be used as proxies of the corresponding full-length proteins [26].

Finally, despite its relatively low phosphatase activity, TULA-1 is capable of dephosphorylating ZAP-70, a protein tyrosine kinase (PTK) critical for TCR/CD3 signaling [26,28,37], although the lack of TULA-2 alone influences ZAP-70 phosphorylation to a much greater extent than the lack of TULA-1 alone [28]. Other substrates of TULA-1 are not known and are hard to predict due to the lack of comprehensive substrate specificity data for this family member [29]. It was also suggested that TULA-1 has PTP-independent functions, which may be involved in T-cell death [38,39] and activation [21], the downregulation of TCR/CD3 [16], chromosome segregation [19] and HIV-1 production [18]. 

## 3. Regulatory Effect of TULA-2 on Platelet Signaling and Activation

### 3.1. Effects of TULA-Family Proteins in Cells Other Than Platelets

Although this review is focused on platelets, it should be noted that TULA-family proteins play a regulatory role in several different cell types. Originally, it was shown that both TULA-1 and TULA-2 downregulate signaling through TCR/CD3 and the resulting T-cell proliferation and cytokine secretion [3,12,28]. The absence of TULA-family members has been shown to upregulate TCR/CD3 signaling, elevate T-cell responses and exacerbate inflammation in a mouse trinitrobenzene sulfonic acid-induced colitis model [37]. In all these studies, the lack of both TULA-1 and TULA-2 in dKO exerted a higher effect on T-cell responses than the lack of either single family member.

TULA-1 and TULA-2 also downregulate the signaling, activation and responses of monocytes and dendritic cells (DCs), important immune cells belonging to the myeloid lineage. Thus, dKO bone marrow (BM) monocytes and BM-derived DCs show an increase in antifungal activity toward *Candida albicans*, a yeast pathogen, as well as in signaling mediated by Dectin-1, a receptor for β-glucan, a major component of the fungal cell wall [40]. The effect of TULA proteins appears to be cell-type-specific, since dKO mouse neutrophils, which are also a myeloid cell type, show a decrease in their antifungal activity [40]. Furthermore, BM-derived monocytes from TULA-1/TULA-2 dKO mice exhibit an increase in antibacterial activity and interferon (IFN)-γ production in response to *Francisella tularensis* [41].

Consistent with the effects of TULA proteins on myeloid immune cells, the differentiation and physiological functions of osteoclasts, which are specialized bone-resorbing cells of the macrophage lineage, are likewise downregulated by TULA-2 [42]. This study demonstrated a substantial increase in signaling through the Fcγ receptor in TULA-2-deficient BM-derived macrophages, which were used as a model of osteoclasts. TULA-2 has also been identified in the protein complex associated with the Syk PTK in basophilic/mast cells stimulated through the FcεRI receptor [43]. In these cells, TULA-2 downregulated FcεRI-mediated signaling and transcription, as well as degranulation, a key biological response of these cells.

For systems in which the molecular basis of these effects was examined [3,12,28,37,40,42,43], a common theme emerged: TULA-family proteins downregulated signaling through receptors bearing an immunoreceptor tyrosine-based activation motif (ITAM) as an essential signaling structure, which, upon its phosphorylation, interacts with the Syk-family PTKs Syk and ZAP-70, thus triggering the phosphorylation and activation of these PTKs and subsequent downstream signaling dependent on their kinase activity [44,45,46,47]. ZAP-70 and Syk have been identified as regulatory targets of TULA-dependent dephosphorylation in this type of signaling pathway in various cells [3,12,28,37,40,42,43]. This theme is also evident from the findings obtained in experiments with platelets and is discussed in detail below.

### 3.2. Effects of TULA-2 on Signaling Mediated by the Glycoprotein VI (GPVI) Collagen Receptor

It has been shown that ubiquitously expressed TULA-2 is substantially overexpressed in platelets as compared to its expression in peripheral blood mononuclear cells [48]. The high platelet expression level of TULA-2 correlates with the high level of TULA-2 transcriptional upregulation demonstrated in the course of human megakaryocyte development in vitro [49]. Together with the finding that Syk is a key PTK in platelet signaling [50,51,52,53,54,55,56,57] and a bona fide cellular substrate of TULA-2 [27,29], the high expression of TULA-2 in platelets renders this PTP a critical player in the system of platelet signaling regulation.

**Figure 2:**
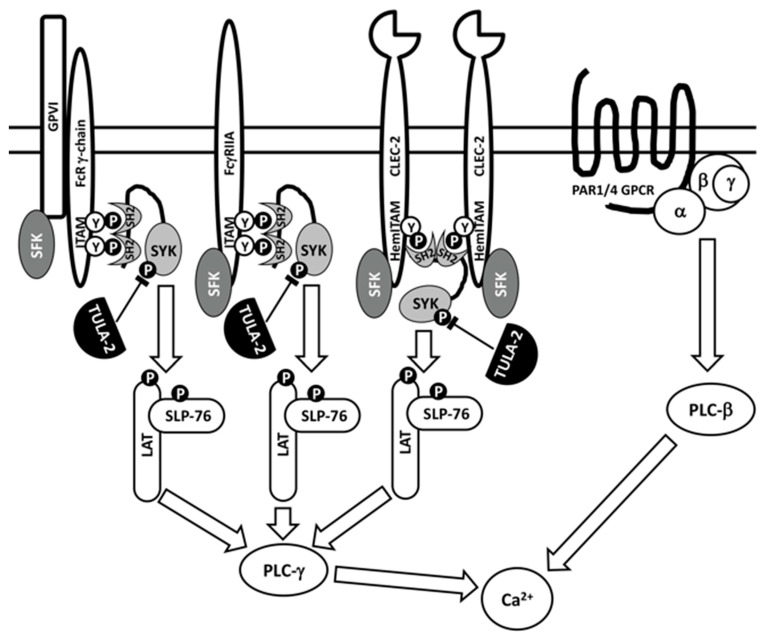
TULA-2 downregulates platelet signaling mediated by Syk. Major upstream events of platelet signaling through immunoreceptor tyrosine-based activation motif (ITAM)- or hemITAM (hemi-ITAM)-bearing receptors and G-protein-coupled receptors (GPCR) are schematically represented. Early signaling events through ITAM- or hemITAM-bearing receptors involve Src-family protein tyrosine kinases (SFKs) and Syk, a protein tyrosine kinase interacting with phosphotyrosines of ITAMs and hemITAMs through its tandem SH2 domains (see details in the text). This scheme illustrates how Syk, following activation through a receptor, phosphorylates its protein substrates, including LAT and SLP-76 adaptors, which interact with other signaling proteins activating PLC-γ, thus increasing the intracellular Ca^2+^ concentration. Activation through the protease-activated receptor (PAR), which is a GPCR, increases Ca^2+^ in a Syk-independent fashion. TULA-2 downregulates Syk-mediated receptor signaling by dephosphorylating Syk phosphotyrosines, which positively regulate activity of this kinase. Various events dependent on receptor-induced Syk activation are downregulated by TULA-2, not only those depicted in this figure.

Initial studies of the effects of TULA-family proteins on platelet activation were conducted using TULA-1/TULA-2 dKO mice lacking both TULA-1 and TULA-2 [48], because both family members had previously been shown to be critical for signaling regulation in T cells [3]. Signaling through the glycoprotein VI (GPVI)/Fc receptor-γ chain (FcR-γ chain) complex, the primary platelet receptor of collagen, which transduces signals through an ITAM located in the cytosolic tail of FcRγ, was dramatically facilitated in TULA-1/TULA-2 dKO platelets; Syk and PLCγ2 showed hyperphosphorylation on tyrosine, the kinase activity of Syk was elevated, and Ca^2+^ mobilization was enhanced. Physiological responses in vitro, such as aggregation and dense granule secretion, were also enhanced in dKO platelets. In contrast, signaling through protease-activated receptor 4 (PAR4), a G-protein-coupled receptor of thrombin, was not altered in dKO platelets (Figure 2). Finally, both the tail bleeding time and the time to carotid artery occlusion in an FeCl_3_-induced thrombosis model were significantly reduced, and the thrombi formed were substantially more stable in TULA-1/TULA-2 dKO mice than in WT mice [48]. Overall, these data indicated that the lack of TULA-family proteins specifically facilitated GPVI/FcRγ-mediated signaling and functional responses in platelets.

The use of dKO mice raised a question of the relative contributions of TULA proteins to the observed effects. While TULA-2 is greatly overexpressed in platelets as compared to other cells, TULA-1 is not detected in platelet lysates using Western blotting [48]. Proteomics reveals the presence of TULA-1 in mouse platelets, but at a level ~8- and 10-fold lower than those of TULA-2 in mice and humans, respectively [58,59]. Since the level of TULA-1 is much lower than that of TULA-2 and since the PTP activity of TULA-1 is significantly lower than that of TULA-2, it was reasonable to conclude that the effect of TULA-1/TULA-2 dKO is primarily attributed to TULA-2 KO. Indeed, TULA-2 KO platelets showed GPVI-induced Syk phosphorylation, aggregation and secretion that were very similar to those demonstrated by dKO platelets, whereas the responses of WT and TULA-2 KO platelets to GPVI agonists were indistinguishable [60]. Hence, TULA-1 at its physiological level appears not to affect platelet responses, and TULA-2 is by far the major if not the sole TULA-family regulator in platelets. Consistent with this conclusion, subsequent studies of the TULA-mediated regulation of platelets have been conducted in TULA-2 KO systems.

### 3.3. Effects of TULA-2 on Signaling through FcγRIIA, A Receptor for the Fc Fragment of IgG

Signaling through the GPVI/FcRγ complex is not the only target of TULA-2-mediated negative regulation in platelets. Signaling through FcγRIIA, an ITAM-bearing receptor for the Fc fragment of IgG, is also downregulated by TULA-2 [61,62] (see Figure 2). In these studies, which were conducted with platelets from transgenic mice expressing human FcγRIIA, a decrease in the TULA-2 level achieved using various approaches upregulatedtyrosine phosphorylation of Syk and other signaling proteins, integrin activation, Ca^2+^ mobilization and platelet aggregation in response to both GPVI- and FcγRIIA-mediated signaling. Consistent with the initial study [48], these results indicated that TULA-2 failed to regulate platelet activation in response to thrombin [61,62]. Therefore, one can conclude that TULA-2 specifically regulates ITAM-mediated signaling, which, in platelets, depends on the functions of Syk.

### 3.4. Effects of TULA-2 on Signaling through the C-Type Lectin-like (CLEC)-2 Receptor

Recent studies also demonstrated the negative regulatory effect of TULA-2 on CLEC (C-type lectin-like receptor)-2-mediated platelet activation [63]. The CLEC-2 receptor bears HemITAM (for hemi ITAM), a YXX(L/I) sequence representing one-half of an ITAM, and it has been speculated that signaling through HemITAMs occurs due to the binding of the Syk tandem SH2 domains to two juxtaposed phosphorylated HemITAMs [64] (see Figure 2). TULA-2 KO platelets exhibit an increase in tyrosine phosphorylation of Syk and other signaling proteins, thromboxane production, aggregation and secretion in response to CLEC-2 agonists [63] in a manner consistent with the effects of TULA-2 on ITAM-mediated signaling.

### 3.5. Physiological Consequences of TULA-2-Mediated Signaling Regulation

Importantly, the studies discussed above definitively demonstrate the significant effects of TULA-2 in vivo. A reduced level of TULA-2 is associated with a shortened tail bleeding time [48,62], enhanced FeCl_3_-injury-induced thrombosis [48] and an exacerbated heparin-induced thrombocytopenia (HIT)-like reaction [61,62] in mice. Together with an inverse correlation between the level of TULA-2 in human platelets from multiple donors and these platelets’ in vitro responses to anti-CD9, which models platelet stimulation in the context of HIT [61], these results indicate that the effects of TULA-2 on platelets are highly relevant for both normal physiological and pathological platelet activation. Notably, even the moderate modulation of the TULA-2 level is sufficient to exert a detectable effect on platelets; a two-fold decrease in the TULA-2 expression level in heterozygous KO/WT mice [62] and its differential expression in human individuals by approximately the same factor [61] are linked to a significant difference in platelet responses.

The specific physiological consequence of the downregulation of ITAM-mediated signaling by TULA-2 remains to be fully understood. It is likely that this regulation acts primarily as a biological brake preventing platelet responses to sub-optimal stimuli. This notion is consistent with several observations: (i) TULA-2 inhibits platelet responses to a much greater extent at low than at high agonist concentrations [60,61,62,63], (ii) TULA-2 inhibits platelet signaling at early time points [29,61,63], (iii) Syk pY346, an early (and possibly the earliest) phosphorylated regulatory site of Syk appears to be the best substrate site of TULA-2 [29,60] (see below for a detailed discussion). However, TULA-2 strongly inhibits platelet receptor signaling at late time points, as well [29,60,61,62,63]. This finding suggests that TULA-2 may also facilitate the return of a platelet to its quiescent state if not all checkpoints on the platelet’s path from this quiescent, non-adhesive patrolling state to the fully activated pro-adhesive state that ensures hemostasis have been passed [65].

## 4. Molecular Basis of the Regulatory Effect of TULA-2 on Platelet Signaling and Activation

**Figure 3:**
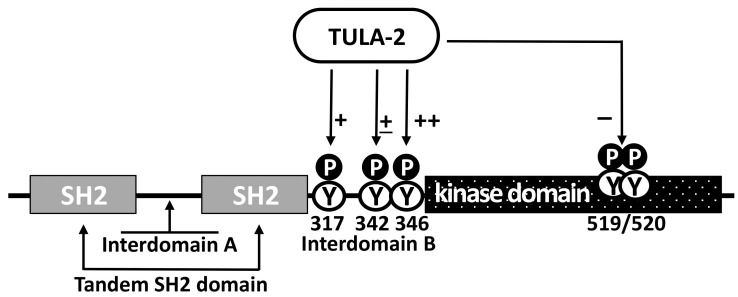
Effect of TULA-2 on Syk regulatory phosphotyrosines. Major domains, interdomain regions and regulatory phosphotyrosines (pY) of Syk are depicted (residue numbering is for mouse Syk). pY342 and pY346 exert positive regulatory effects on Syk, while pY317 is a negative regulatory site. The pY519/pY520 site is located in the activation loop of Syk and represents a marker of Syk activation. The differential ability of TULA-2 to dephosphorylate the sites depicted here is indicated and varies from very strong (++) to strong (+) to moderate (±) to the lack thereof (−). See the text for detail.

The correlation of the downregulatory effects of TULA-2 on physiological platelet responses with the TULA-2-dependent dephosphorylation of Syk, together with the specificity of these effects for Syk-dependent platelet responses when ITAM-mediated signaling is affected, while G-protein-mediated signaling is not, strongly suggests that Syk is the main regulatory target of TULA-2 [48,61,62]. It has been demonstrated that the level of phosphorylation on Syk Y346, Y317, and Y519/Y520 sites, which are known to be phosphorylated in response to receptor stimulation in various cell types [66,67,68,69,70], is significantly reduced in WT platelets as compared to platelets from TULA-2 KO or TULA-1/TULA-2 dKO mice [29,48] (Figure 3). A decrease in pY519/pY520, a major activation marker of Syk [71,72], is likely a consequence of a decrease in the phosphorylation of Syk regulatory sites directly targeted by TULA-2, since Syk kinase activity is thought to be affected by multiple pY-sites [45,47,68,69,73].

The effects of TULA-2 on Syk activity may be complex, because not only Syk pY346 but also, to some extent, Syk pY342 has been shown to be targets of TULA-2 [60], while both of them profoundly regulate Syk activity [60,66,67,73,74,75,76,77]. Based on the results obtained with other cell types, the effects of both pY342 and pY346 on Syk activity are positive [66,67,74,75,76], but in platelets, this issue has not been addressed in detail. Additionally, pY346 and pY342 appear to functionally interact [60], and this interaction may introduce additional complexity to the pY-dependent regulation of Syk activity. Furthermore, pY317 also appears to be a TULA-2 target while negatively regulating Syk by virtue of being a key binding site for Cbl, a negative regulator of Syk activity [78,79] (see Figure 3). Notably, the molecular basis of the Cbl-mediated regulation of Syk differs in platelets and nucleated cells. In nucleated cells, Cbl acts by inducing the ubiquitylation and subsequent degradation of phosphorylated and, hence, activated Syk, thus reducing Syk activity in the cell [33,35,79,80]. In platelets, Syk binds to Cbl and becomes ubiquitylated, but its degradation does not occur; this result led to the speculation that Cbl downregulates platelet Syk by facilitating the interaction of phosphorylated Syk with a protein tyrosine phosphatase [34]. In light of the subsequent progress, it is possible that TULA-2, which binds to both Syk and Cbl as well as to ubiquitin [5,17,48], may act as such a phosphatase. Overall, the regulation of Syk by TULA-2 is expected to be very complex, since the functions of Syk depend on multiple pY-sites, several of which are targeted by TULA-2, as indicated above. The opposite effects of some of these sites on Syk activity make the effects of TULA-2 on the functions of Syk particularly intricate.

Despite the established importance of the TULA-2-dependent dephosphorylation of Syk for platelet regulation, it cannot be ruled out that TULA-2 may dephosphorylate other protein substrates, for example, Src-family kinases (SFKs). The dephosphorylation of Src by TULA-2 has been shown in reaction mixes and in 293T cells overexpressing both Src and TULA-2 [12]. Likewise, the dephosphorylation of Fynby TULA-2 has been shown in vitro [27]; in this case, the dephosphorylation of Fyn appeared to be specific, since neither Lck nor Yes (the other SFKs examined) exhibited detectable dephosphorylation by TULA-2. The rate of the TULA-2-dependent dephosphorylation of Fyn shown in this study appeared to be substantially less than that of Syk [27]; this finding matches well with the difference between the pY-sites of Syk and Fyn with regard to their TULA-2 substrate specificity determinants. Syk pY346 and, to a slightly lower extent, Syk pY317 are predicted to be very good substrate sites for TULA-2; Syk pY342 is predicted to be less advantageous based on substrate specificity determinants, and these predictions are validated by experiments with protein mixes and activated platelets [29,60]. In contrast, the major pY-sites of Fyn (and SFKs, in general) are predicted to be poor substrates of TULA-2 [29,60]. However, dephosphorylation in the cellular context depends not only on the kinetic constants governed by substrate specificity determinants but also on the concentration and localization of TULA-2 and its potential substrate, so even sub-optimal substrates may be dephosphorylated. Notably, the effect of the TULA-2-driven dephosphorylation of SFKs in platelets, if it occurs, would exert a global effect on their signaling and responses, including Syk phosphorylation and activation, since SFK activity is essential for triggering all ITAM-mediated events (see Figure 2) and is involved in many other diverse signaling pathways and regulatory circuits in platelets and other blood and immune cells [47,81,82,83,84,85,86,87,88]. Whether or not other substrates of TULA-2 exist in platelets remains unclear. Although this is possible, it should be noted that most of the tyrosine-phosphorylated protein material from GPVI-stimulated platelets that binds to the inactivated substrate-trapping form of TULA-2 [89] corresponds to Syk [60], while the amounts of other tyrosine-phosphorylated proteins bound to this reagent are low [60], suggesting that Syk is the major substrate of TULA-2 in platelets.

Another potentially important molecular element of the TULA-2-mediated regulation of platelet activation is ubiquitylation, since (i) UBA of TULA-family proteins is capable of binding ubiquitin [4,5], (ii) ubiquitylated pY-containing proteins are accumulated in TCR/CD3-activated TULA-1/TULA-2 dKO T cells [36], (iii) mutations in the UBA domain reduce the ability of TULA-2 to reconstitute WT signaling levels in dKO T cells [12] and (iv) the contribution of the Cbl-mediated ubiquitylation of Syk to the regulation of this PTK in platelets has been considered [34]. Hence, the binding of TULA-2 to ubiquitylated Syk has been postulated in the computational model of platelet activation, which predicts the time course of Syk phosphorylation in response to GPVI-mediated stimulation quite well [90]. However, the contribution of Syk ubiquitylation to its TULA-2-dependent dephosphorylation in platelets has not been experimentally established. Thus, non-ubiquitylated Syk is bound to TULA-2 in cells overexpressing them and is dephosphorylated by TULA-2 in the cells and in the mix of recombinant proteins [27,48].

## 5. Regulation of TULA-2 Level and Activity in Platelets

Considering the importance of TULA-2 as a regulator of platelet functions, the mechanisms controlling the effects of TULA are of great interest. It should be noted that the function of TULA-2 as a PTP can be regulated simply by the availability of substrates; fully active TULA-2 may be present in the cell but exert no substantial effect until a pY-site with the characteristic TULA-2 specificity determinants is formed on a protein accessible to TULA-2, such as the pY346 site of Syk, as a result of specific receptor-mediated signaling. However, the existence of mechanisms regulating the TULA-2 amount and/or specific activity could substantially enrich the flexibility of this regulatory circuit. One such mechanism is mediated by microRNA; it has been shown that miR-148a targets the 3′ untranslated region of TULA-2 mRNA and downregulates the level of TULA-2 in platelets and erythroleukemia cells, thus facilitating ITAM- and Syk-dependent signaling through the FcγRIIA receptor [61]. The treatment of cell line cultures or mice with anti-miR-148a elevates the level of TULA-2 and suppresses receptor-mediated signaling and activation as well as platelet-dependent thrombotic events in vivo. These results suggest that anti-miR-148a-type reagents can be used, in principle, as a therapy against thrombosis, but this possibility remains to be examined further.

The functions of TULA-2 may be affected by its posttranslational modifications, as well. The ability of TULA-family proteins to bind to ubiquitin appears to be critical for the TULA-dependent inhibition of epithelial growth factor receptor degradation [4,5,17], and hence, the mono-ubiquitylation of TULA-family proteins themselves downregulates this effect by inducing an intramolecular interaction between the TULA UBA domain and a ubiquitin residue attached to TULA [5]. However, the contribution of the ubiquitylation-mediated regulation of TULA to platelet activation has never been demonstrated.

Finally, the phosphorylation of TULA-2 on a tyrosine residue in the N-terminal region in stimulated platelets has been reported [91]. This pY-site appears to be conserved, since it has previously been detected in stimulated T cells [92]. Furthermore, several serine/threonine phosphorylation sites have been identified in TULA-2 in platelets [91,93]. Phosphorylation is generally recognized as a powerful and widely employed biological mechanism regulating protein activity. However, the role of phosphorylation in the regulation of TULA-family functions is currently obscure not only in platelets but also in general. Overall, it remains poorly understood how the functions of TULA-family proteins are regulated and whether this regulation plays a significant role in the effect of TULA-2 on platelet signaling and activation.

## 6. Conclusions

The available data allow us to conclude that TULA-2 is a critically important regulator of platelet signaling mediated by ITAM- or hemITAM-bearing receptors and the platelet responses induced by this signaling. Furthermore, it has been convincingly demonstrated that TULA-2 exerts significant effects on platelet functions in vivo. The molecular basis of this effect is provided by a high level of TULA-2 expression in platelets, far exceeding that in other blood/immune cells, together with the substrate specificity of TULA-2 PTP activity rendering Syk, a key PTK of platelet ITAM/hemITAM-mediated signaling, a prime target of TULA-2. The molecular mechanisms of TULA-2′s effects on Syk-mediated events clearly involve the dephosphorylation of specific regulatory pY-sites of Syk, including Syk pY346, although the dephosphorylation of pY-sites on other proteins might also participate in the events of TULA-2-mediated regulation. The effects of TULA-2 appear to be regulated at the TULA-2 transcript level, although the phosphorylation-mediated regulation of TULA-2 cannot be ruled out.

## Data Availability

Not applicable.
